# Fast diagnosis of sporotrichosis caused by *Sporothrix globosa*, *Sporothrix schenckii*, and *Sporothrix brasiliensis* based on multiplex real-time PCR

**DOI:** 10.1371/journal.pntd.0007219

**Published:** 2019-02-28

**Authors:** Mingrui Zhang, Fuqiu Li, Ruoyu Li, Jie Gong, Fei Zhao

**Affiliations:** 1 Department of Dermatology, the Second Hospital of Jilin University, Changchun, Jilin Province, China; 2 Department of Dermatology, Peking University First Hospital, Beijing, China; 3 Beijing Key Laboratory of Molecular Diagnosis on Dermatoses, Beijing, China; 4 National Institute for Communicable Disease Control and Prevention, Chinese Center for Disease Control and Prevention, State Key Laboratory of Infectious Disease Prevention and Control, Beijing, China; University of Tennessee, UNITED STATES

## Abstract

The accurate diagnosis of sporotrichosis and identification at the species level are critical for public health and appropriate patient management. Compared with morphological identification methods, molecular diagnostic tests are rapid and have high sensitivity and standardized operating processes. Therefore, we designed a novel multiplex real-time polymerase chain reaction (PCR) method based on the *calmodulin* (CAL) gene for the identification of clinically relevant *Sporothrix* species: *S*. *globosa*, *S*. *schenckii s*. *str*., and *S*. *brasiliensis*. We evaluated the assay with clinical and spiked samples and assessed its diagnostic performance by comparing the results to those of culture and species-specific PCR. Thirty-three DNA templates were used to detect assay specificity, and three plasmids were constructed to create a standard curve and determine the limits of detection (LODs). For nucleic acid detection, the sensitivity and specificity reached 100%. The LODs were 10 copies, 10 copies and 100 copies for *S*. *globosa*, *S*. *schenckii s*. *str* and *S*. *brasiliensis*, respectively. For the clinical samples, the positive detection rates by culture, species-specific PCR and the multiplex real-time PCR assay were 87.9% (29/33), 39.4% (13/33), and 93.9% (31/33), respectively. For the spiked samples, the positive detection rates were both 100% for *S*. *schenckii s*. *str* and *S*. *brasiliensis*. Based on the above results, compared with culture and other molecular diagnosis methods, the novel multiplex real-time PCR assay is effective, fast, accurate, and highly sensitive. It has a lower reaction cost and lower sample volume requirements, can detect co-infections, and allows for standardized operation and easier interpretation of results. In the future, this assay could be developed into a commercial kit for the diagnosis and identification of *S*. *globosa*, *S*. *schenckii s*. *str*, and *S*. *brasiliensis*.

## Author summary

Sporotrichosis is a subacute or chronic infectious disease caused by dimorphic fungi of *Sporothrix* spp. The genus *Sporothrix* consists of several species with different geographic distributions, virulence, and antifungal susceptibilities, making species-level identification necessary. *S*. *brasiliensis*, *S*. *globosa*, *S*. *schenckii s*. *str* and *S*. *luriei* make up the “pathogenic clade” of the genus *Sporothrix*. Importantly, *S*. *luriei* has a low clinical-epidemiological impact within this genus. Therefore, we designed a novel multiplex real-time PCR method using fluorescent probes for the identification of *S*. *globosa*, *S*. *schenckii s*. *str*, and *S*. *brasiliensis*. We designed a pair of primers based on the conserved sequence of the *calmodulin* gene of *Sporothrix* spp. and probes with different fluorescent signals based on the divergent sequences of *S*. *globosa*, *S*. *schenckii s*. *str* and *S*. *brasiliensis*. Through the verification of nucleic acid, clinical and spiked sample detection, the multiplex real-time PCR could quickly and accurately identify the three clinically relevant species of *Sporothrix* spp. with high sensitivity. This new assay could be applied in epidemiology, clinical diagnosis, and experiments with sporotrichosis to control new outbreaks, reduce diagnostic and identification time, and improve test efficiency.

## Introduction

Sporotrichosis is a subacute or chronic infectious disease caused by dimorphic fungi of *Sporothrix* spp., which are distributed worldwide, especially in tropical and subtropical regions[1–2]. The infection generally occurs through traumatic inoculation of contaminated plant debris[3–4] or through bites and scratches from infected animals, mostly felines[5–6]. The disease can occur sporadically or in outbreaks[7–8]. The primary lesion is usually restricted to the skin and subcutaneous tissue but can subsequently affect adjacent lymphatic vessels[9]. Rarely, this fungus can disseminate through the blood or the lymphatic system and eventually lead to a systemic infection[3]. The pathogen of sporotrichosis, *S*. *schenckii sensu lato*, was recognized as the sole agent for more than a century following its first isolation in 1898 by Benjamin Schenck[10]. However, based on macroscopic characteristics and physiological and molecular aspects, *S*. *schenckii sensu lato* is considered to be a complex of several distinct species, including *S*. *brasiliensis*, *S*. *mexicana*, *S*. *globosa*, and *S*. *schenckii s*. *str*.[11], *S*. *luriei*[12], *S*. *pallida*[13–14], and *S*. *chilensis*[15]. Further, *S*. *globosa*, *S*. *schenckii s*. *str*, *S*. *brasiliensis* are medically important species within the *Sporothrix* genus. These species differ in ecology, distribution and epidemiology. Furthermore, widespread variations in virulence and antifungal susceptibility among these species have been demonstrated. *S*. *brasiliensis*, which is associated with severe clinical forms of sporotrichosis[16], is considered the most virulent species, followed by *S*. *schenckii s*. *str*. and *S*. *globosa*. Therefore, identification at the species level is critical for public health and appropriate patient management[17,18].

Sporotrichosis can be diagnosed through a combination of clinical manifestation and epidemiological and laboratory tests, including direct examination, culture, histopathological examination, molecular detection, sporotrichin skin tests and antibody detection[18]. It is difficult to detect the parasitic budding yeast cells by direct examination, likely because they are too small (2 to 6 μm in diameter). Yeast cells can be observed in tissue by histopathological examination using Haematoxylin and eosin (H&E), Gomori methenamine silver (GMS) or Periodic acid-Schiff (PAS) stain[1]. However, neither direct examination nor histopathological examination can identify the pathogen at the species level. The “gold standard” for diagnosing sporotrichosis is based on conventional culture of clinical specimens obtained from active lesions, pus, secretions or biopsies. After culture on Sabouraud agar (SDA) for 5 to 7 days at 28°C, filamentous hyaline colonies start to grow and then develop a dark colour, especially from the centre of the colonies[19] Positive cultures provide the strongest evidence for sporotrichosis, and isolates obtained from culture can be tested for antifungal susceptibility and phenotypic characterization.

With the development of molecular biology, an increasing number of methods based on nucleic acid detection have been applied for the rapid diagnosis of infectious disease. Many molecular diagnostic tests, including DNA sequencing of “barcoding” genes[20–23], nested PCR[24–25], PCR fingerprinting[26], restriction fragment length polymorphism (RFLP) of different gene targets[27], random amplified polymorphic DNA (RAPD)[7], amplified fragment length polymorphism (AFLP) [8], rolling circle amplification (RCA) [28] and species-specific primers[29], have been developed for *Sporothrix* spp. detection. However, there are still some shortcomings, such as time-consuming procedures (PCR sequencing for at least 12 h); complicated operation steps, which can increase the chance of contamination (nest PCR); insufficient sensitivity (RCA, 3 × 10^6^ copies); and so on. Most of them can only identify isolates from culture, and only a few methods have been evaluated with clinical samples[24–25]. In addition, none of the above methods can detect co-infection simultaneously.

In the present study, we developed a novel multiplex real-time PCR assay to identify the mainly clinical pathogenic species *S*. *globosa*, *S*. *schenckii s*. *str*. and *S*. *brasiliensis*, and we evaluated the assay with clinical and spiked samples.

## Results

### Specificity, standard curve and limits of detection (LODs)

The analytical specificity was examined using 33 DNA templates, including from fungi (28), bacteria (3), a human (1) and a mouse (1) (S1 Table). None of the 33 templates yielded positive signals in the assays; furthermore, nonspecific amplification was not detected. Excluding the negative controls, all 25 *Sporothrix* templates, including *S*. *globosa* (21), *S*. *schenckii s*. *str*. (3) and *S*. *brasiliensis* (1), yielded positive signals, and the positive detection rate for the nucleic acid templates of the assay was 100%. Standard curves (Ct vs. log CFU) for *S*. *globosa*, *S*. *schenckii s*. *str*. and *S*. *brasiliensis* were constructed using the plasmid DNA template by serial 10-fold dilution (S1 Fig). In addition, the results indicated that the LODs of this assay were 10 copies, 10 copies and 100 copies for *S*. *globosa*, *S*. *schenckii s*. *str*. and *S*. *brasiliensis*, respectively.

### Mixed template detection and comparison between multiplex and single fluorescence

For the mixed templates, the multiplex real-time PCR assay could detect the corresponding fluorescent signals in the same tube without nonspecific amplification. The Ct values of different amount templates are shown in Table 1.

**Table 1:** Ct values of different mixed templates detected by multiplex real-time PCR assay.

	Composition of template (copies)^#^				Ct values		
		SG	SB	SS	SG (FAM)	SB (CY5)	SS (VIC)
Single template		**10**^**2**^	/	/	**34.32**	/	/
		/	**10**^**3**^	/	/	**35.64**	/
		/	/	**10**^**2**^	/	/	**35.92**
Mixed template	1:10*	**10**^**2**^	10^3^	10^3^	**35.64**	36.12	32.07
		10^4^	**10**^**3**^	10^4^	29.23	**36.33**	28.41
		10^3^	10^3^	**10**^**2**^	31.12	35.71	**36.35**
	1:100	**10**^**2**^	10^4^	10^4^	**36.9**	32.06	29.03
		10^5^	**10**^**3**^	10^5^	24.05	**37.29**	25.16
		10^4^	10^4^	**10**^**2**^	27.58	32.06	**37.45**
	1:1000	**10**^**2**^	10^5^	10^5^	**38.06**	28.63	25.2
		10^6^	**10**^**3**^	10^6^	21.87	**38.72**	22.52
		10^5^	10^5^	**10**^**2**^	23.94	28.21	**39.09**

Bold font represents detected templates.

* The ratio of detected plasmid and other two plasmids

# SG: *S*. *globosa*; SB: *S*. *brasiliensis*; SS: *S*. *schenckii s*. *str*.

The Ct values of the single fluorescence real-time PCR for *S*. *globosa*, *S*. *schenckii s*. *str*., and *S*. *brasiliensis* were 21.67±0.15, 24.64±0.15, and 27.00±0.11, respectively, while under the same templates and condition, the Ct values obtained from the multiplex real-time PCR were 21.80±0.07, 24.77±0.07, and 27.32±0.08, respectively. There was no significant difference in Ct values between multiplex and single fluorescence real-time PCR except for *S*. *brasiliensis* (t-test, p = 0.03).

### Clinical and spiked sample detection

A total of 40 samples from patients suspected of sporotrichosis were collected (S2 Table). Seven samples were eliminated based on culture and histopathological examination results. The results of the multiplex real-time PCR and species-specific PCR were negative for these 7 samples. Of the 33 selected samples, the positive detection rates of the culture, species-specific PCR and multiplex real-time PCR assays were 87.9% (29/33), 39.4% (13/33), and 93.9% (31/33), respectively (Table 2). The positive detection rates of the culture and multiplex real-time PCR assays were not significantly different (paired χ^2^, p = 0.4142). Differences were observed between the multiplex real-time PCR assay and species-specific PCR (paired χ^2^, p<0.0001).

**Table 2:** The results and positive detection rates of clinical samples by culture, species-specific PCR and multiplex real-time PCR.

	Culture	Species-specific PCR	Multiplex real-time PCR
Positive	29	13	31
Negative	4	20	2
Total	33	33	33
Positive detection rate (%)	87.9	39.4*	93.9

*p<0.0001, compared with multiplex real-time PCR

The isolates from culture were identified by sequencing the CAL gene, and all 29 strains were *S*. *globosa*. Among the 33 samples detected by the multiplex real-time PCR assay, the Ct values of 31 were less than 40 and were judged as positive; the Ct values of 2 samples were greater than 40 and were judged as negative. Only FAM fluorescence was detected; therefore, all of the samples were identified as *S*. *globosa* infections, consistent with the sequencing results from the cultured isolates. Among the 33 samples, 11 were positive by all three methods, and 27 were positive by both culture and multiplex real-time PCR. In addition, culture was positive and multiplex real-time PCR was negative for 2 samples, and culture was negative and multiplex real-time PCR was positive for 4 samples, of which two were also positive by species-specific PCR.

No false positives were detected from the negative control group of spiked samples, and the positive detection rates of *S*. *schenckii s*. *str*., and *S*. *brasiliensis* were both 100% (6/6), while the Ct values were 33.03–38.57 (*S*. *schenckii s*. *str*.) and 30.23–34.84 (*S*. *brasiliensis*).

## Discussion

In 2006, Marimon et al. [21] reported *Sporothrix complex* for the first time, which led to many studies of the differences between species in the *Sporothrix* genus[16–17,22,30–32]. These studies showed the variations between different species and highlighted the need to identify the species level of *Sporothrix* spp.. *Calmodulin* (CAL), *internal transcribed spacer* (ITS), and *elongation factor* (EF), which are recognized as fungal "barcoding" genes, are widely applied in the identification of *Sporothrix* spp.[20–23]. However, all of the methods are based on conventional PCR. Compared to conventional PCR, real-time PCR has many extraordinary advantages, such as rapidity, sensitivity and low risk of contamination. Due to these strong points, real-time PCR is widely used in pathogen detection. However, the application of real-time PCR to *Sporothrix* spp. identification has not yet been reported [18]. In this study, by comparing sequences of the various “barcoding” genes, a target sequence, which can be used to design the primers and probes for three pathogenic species, was found on the *calmodulin* gene of *Sporothrix* spp.. Based on this finding, we established a multiplex real-time PCR to detect *S*. *globosa*, *S*. *schenckii s*. *str* and *S*. *brasiliensis* simultaneously, which not only can improve the sensitivity of *Sporothrix* spp. detection but also could save detection time and costs.

Furthermore, the detection ability of co-infections was assessed by 9 combinations of different amounts of plasmids. The results showed that, when the amounts were different, the amplification of the smaller amount plasmid was affected, and because of the competitive inhibition, the greater the difference in the proportion of plasmids was, the greater the effect was on the Ct value of the small amount of plasmid. For the same template, the Ct values were not significantly different between multiple and single fluorescence for *S*. *globosa* and *S*. *schenckii s*. *str*. Only the intensity of the fluorescent signal was weakened, but this decrease did not affect the judgement of the result. However, a difference in Ct value was observed for *S*. *brasiliensis* (p = 0.03), likely due to the weak amplification efficiency compared to the other two. Considered together, these results demonstrated that the assay was capable of detecting co-infections and that there was almost no mutual interference between the primers and probes in the multiplex system.

In addition, the clinical samples were used to evaluate the performance of the assay compared with the “gold standard” diagnostic and the latest molecular diagnosis methods of culture and species-specific PCR. Culture, as the “gold standard” for sporotrichosis diagnosis, is widely used in clinical practice. However, species-level identification requires further phenotypic identification and physiological tests, which require at least 3–4 weeks. During this period, contamination by fast-growing fungi or bacteria is likely happening. Some patients undergoing antifungal treatment can also have negative culture results. Therefore, histopathological examination results, such as detection of *Sporothrix* spp. yeast cells or typical pathological structures, are also combined with culture results for clinical diagnosis. In this experiment, these diagnostic criteria were followed when the clinical samples were collected. Results were considered negative only if the culture and histopathological examinations both excluded the diagnosis. In the present study, the positive rate of culture was 87.9% (29/33). Of the 4 negative results, 2 samples had contamination from other microorganisms and were judged to be negative, while the multiplex real-time PCR and species-specific PCR results of the 2 samples were both positive. Another 2 samples did not grow after 30 days in culture, while the multiplex real-time PCR results were positive. It is speculated that these 2 cases might have been treated with antifungal therapy or affected by the location of the biopsy. Based on the different lengths of the CAL gene sequences, species-specific PCR[29], reported by Rodrigues et al., could identify *S*. *brasiliensis*, *S*. *schenckii s*. *str*, *S*. *globosa*, *S*. *mexicana*, *S*. *pallida*, and its relative, *Ophiostoma stenoceras*. The reaction conditions for this species-specific PCR included 35 cycles, and amplification could not be detected after 35 cycles. In this experiment, according to the results of the multiplex real-time PCR, which was performed for 45 cycles, most of the Ct values were greater than 35 (22/33). In this situation, the results of species-specific PCR were often negative.

Until now, no reports of *S*. *brasiliensis* have appeared, and there have been only four known isolates of *S*. *schenckii s*. *str*. found in China[33]. To compensate for the singularity of pathogens in clinical specimens, an evaluation of the spiked samples was performed. Yeast cells, the pathogenic phase of *Sporothrix* spp., were selected for mixing with negative tissue samples, and positive results were obtained from all of the spiked samples, which were mixed with different numbers of yeast cells. In addition, the standard curves of assay were established on direct dilution of plasmids, whereas the clinical samples were prepared using DNA extraction kit. Since DNA lost is unavoidable during the extraction process, the sensitivity of the multiplex real-time PCR was applied to the extracted DNA samples, not the original clinical samples. Considering that different extraction methods result in varying degrees of DNA lost, it is recommended to use a method with high yield of DNA for clinical samples extraction.

In conclusion, the novel multiplex real-time PCR assay was effective, fast, accurate, and highly sensitive. It had a lower reaction cost and sample volume requirements, could detect co-infections and allowed for standardized operation and easier interpretation of results. However, the assay must still be validated with clinical samples of *S*. *schenckii s*. *str* and *S*. *brasiliensis*. In the future, the number of clinical samples used to validate the assay must be increased, and the assay could be further verified using pus or secretions from active lesions as the templates.

## Methods

### Isolates and specimens

Twenty-five *Sporothrix* spp. isolates (including 21 *S*. *globosa*, 3 *S*. *schenckii s*. *str*, and 1 *S*. *brasiliensis*), twenty-eight other fungal strains (including agents of superficial, subcutaneous, and systemic mycoses in humans and animals), three bacterial strains, one human genomic DNA sample and one BALB/c-mouse genomic DNA sample were used to develop the PCR assays (S1 Table). The fungi were obtained from the Collection of Pathogenic Fungi at the Research Centre for Medical Mycology, Peking University (BMU, Beijing, China), the bacterial DNA were obtained from the National Institute for Communicable Disease Control and Prevention, Chinese Centre for Disease Control and Prevention (Beijing, China), the human DNA was obtained from a healthy volunteer, and the mouse DNA was obtained from a BALB/c mouse. All of the fungal strains were previously characterized at the species level via morphological analysis and sequence analysis of the rDNA operon (ITS1-5.8S-ITS2) and the CAL gene.

A total of 40 tissue biopsies were collected between September 2017 and August 2018; the clinical data are shown in S2 Table. These samples were collected from patients in the Dermatology Department of the Second Hospital of Jilin University, whose clinical manifestations indicated suspected sporotrichosis. The clinical symptoms of the subjects were examined by professional physicians. In addition, 6 other negative human samples were collected from different volunteers and were used as artificially contaminated samples to simulate clinically infected specimens of *S*. *schenckii s*. *str* and *S*. *brasiliensis*. All of the specimens were skin and subcutaneous tissue harvested by surgery. When the specimens were collected, informed consent was obtained based on the guidelines and agreements of the institutional ethics committee.

### Genomic DNA extraction and plasmid DNA preparation

All of the fungal isolates were subcultured on 2% potato-dextrose agar (PDA) slide medium at 28°C for 7–14 days. All of the tissues were cut into small pieces with sterile scissors, and then all of the pieces were placed in liquid nitrogen and ground thoroughly with a mortar and pestle. DNA was extracted and purified with the QIAamp DNA Mini Kit (QIAGEN, Hilden, Germany) in accordance with the manufacturer’s instructions; detailed steps are provided in the supplemental methods (S1 Text). The quality of the extracted DNA was assessed by amplification of part of the rDNA operon or β-globin using universal primers[34]. The amplified products were visualized via agarose gel electrophoresis and UV detection. A single amplification product indicated that the sample was free of PCR inhibitors.

Three plasmids were constructed to create a standard curve and to determine the LODs of the multiplex real-time PCR assay. The CAL regions of *S*. *globosa* (BMU 09028), *S*. *schenckii s*. *str* (CBS498.86^T^) and *S*. *brasiliensis* (CBS 120339^T^) were cloned into pMD-18T vectors (Takara, Dalian, China). The plasmids were transformed into *E*. *coli* DH5a competent cells, and the cells that contained recombinant plasmids were cultivated in lysogeny broth for 24 h. The plasmids were then extracted from the cultured *E*. *coli* suspension with a Qiagen Plasmid Mini Kit (QIAGEN, Hilden, Germany). Plasmid concentrations were measured with a NanoDrop 2000 spectrophotometer (Thermo Fisher Scientific, USA), and the copy numbers of the plasmids were calculated from their total base lengths and DNA concentrations using the equation of Godornes et al. [35]. The DNA samples and plasmids were stored at -20°C until use.

### Multiplex real-time PCR assay

All of the CAL sequences belonging to *Sporothrix* spp. were selected from the National Center for Biotechnology Information (NCBI) database to develop specific primers targeting the conserved sequence of *Sporothrix* spp. and probes marked by different fluorescent signals targeting the divergent sequences of *S*. *globosa*, *S*. *schenckii s*. *str* and *S*. *brasiliensis* (details in Table 3). Primer Express software (version 3.0; Life Technologies-Applied Biosystems) was used to design the primers and probes and to evaluate melting temperatures, GC content, dimers, and mismatches in the candidate primers and probes.

**Table 3:** Primers and probes used in the multiplex real-time PCR assay.

Primer/probe name	Sequence (5’-3’)
Spo-F	CATTGACTTCCCTGGTAYGTTTGAC
Spo-R	CARGAACTCTGTGGAYGGTTAGC
Spo-MGB-SGP	FAM- AGCACGGGTAGACAT -MGB
Spo-MGB-SSP	VIC- CTGCACTATGACACGGT -MGB
Spo-MGB-SBP	CY5- ACACACGGTTATCC -MGB

Each PCR mixture consisted of 2.5 μL of 10x Platinum Buffer (Life Technologies-Invitrogen), 4.0 μL of MgCl_2_ (50 mM), 0.2 μL of each primer (25 μM), 0.1 μL of each probe (25 μM), 0.25 μL of Platinum Taq DNA polymerase (5 U/μL; Life Technologies-Invitrogen), 1.5 μL of PCR nucleotide mix (10 mM), 5 μL of DNA template, and nuclease-free water to achieve a final volume of 25 μL. Multiplex real-time PCR was performed in a CFX96 Real-time PCR Detection System (Bio-Rad, Hercules, CA, USA) under the following conditions: predenaturation at 95°C for 3 min, followed by 45 cycles of 95°C for 15 s and 60°C for 30 s. The data were analysed with CFX Manager software (version 3.1; Bio-Rad).

### Analysis of specificity, standard curves and LODs

The analytical specificity of the assays was tested by analysing 33 DNA samples derived from other pathogenic fungi, bacteria and tissues from a human and a mouse. The analytical sensitivity, standard curves and LODs of the assays were determined by using three 10-fold dilutions of the previously constructed plasmids, ranging from 2.0×10^5^ copies/μL to 0.2 copies/μL. *S*. *globosa*, *S*. *schenckii s*. *str* and *S*. *brasiliensis* were detected by FAM, VIC, and CY5 fluorescence, respectively. The detection limit was noted for each probe. Each dilution of the plasmids was assayed in triplicate.

### Analysis of mixed template detection and comparison between multiplex and single fluorescence

The detection ability for a mixed template was determined by analysing the Ct values from 9 compositions of plasmid mixtures. The amount of detected plasmid was set at one gradient larger than LOD (i.e., 100 copies for *S*. *globosa*; 1000 copies for *S*. *brasiliensis*; 100 copies for *S*. *schenckii s*. *str*). The amount of the other two plasmids were 10-fold, 100-fold, and 1000-fold greater than that of the detected plasmid. Each template mixture was comprised of three plasmids of different proportions (details in Table 1). The mixed templates were detected by multiplex real-time PCR. The obtained Ct values were compared with those of single plasmid detection in the same reaction system under the same conditions. The detection ability of the multiplex and single fluorescence assays was tested by comparing the Ct values from four different reaction systems (one multiplex and three single fluorescence values of FAM/VIC/CY5) with the same templates under the same conditions. Each reaction was assayed in triplicate.

### Evaluation of multiplex real-time PCR with clinical and spiked samples

A total of 40 specimens from biopsies were collected, and each was divided into three parts. One part was used for culture (PDA, 28°C), one for histopathological examination (HE and PAS) and one for DNA extraction. The performance of the multiplex real-time PCR assay was evaluated by comparison with the culture method and the species-specific PCR[29]. After 4 weeks of culture, no fungal growth or growth of contaminating microorganisms was judged as negative[18]. The results of the histopathological examination showed a mixed suppurative and granulomatous inflammatory reaction in the dermis and subcutaneous tissue, and the detection of asteroid bodies or *Sporothrix* spp. yeast cells by PAS was suggestive of sporotrichosis. The clinical diagnosis was made by combining the results of the culture and histopathological examination.

To evaluate the assay detection ability for *S*. *schenckii s*. *str* and *S*. *brasiliensis* infectious samples, we used artificially contaminated (spiked) samples to simulate clinically infected specimens. Each of the 6 negative human samples was divided into three parts, two of which were used to simulate infected specimens and one of which was used for negative controls. *S*. *schenckii s*. *str* (CBS498.86^T^) and *S*. *brasiliensis* (CBS 120339^T^) were subcultured on brain heart infusion (BHI) agar medium and incubated at 35°C for 7 days to obtain the yeast cells of *Sporothrix* spp. The yeast cell suspensions of *S*. *schenckii s*. *str* and *S*. *brasiliensis* were prepared with sterile saline solution, and the OD was adjusted at 520 nm to 0.2, approximately corresponding to a concentration of 10^6^ cells/mL.[28]. Then, 10, 50, 100 μL each of the suspensions were mixed with 2 different negative human samples, respectively. DNA extraction procedures were the same as described above.

A no-template control (NTC), a negative control (NEG) and a positive control (POS) were established for each test of the multiplex real-time PCR assay. When the amplification result showed NTC (-), NEG (-), and POS (+), the test was considered a valid amplification. A Ct value from the valid amplification of less than 40 was judged as positive; otherwise, it was negative. The species-specific PCR was performed according to the literature[29], and a single clear band shown by gel electrophoresis and UV detection was judged as positive; otherwise, it was negative.

### Statistical analysis

The quantitative data are presented as the mean ± standard deviation (SD). The differences in Ct values between multiplex and single fluorescence were tested with the independent samples t-test. The differences in positive detection rates between the multiplex real-time PCR and culture and between the multiplex real-time PCR and species-specific PCR were tested with the paired chi-square test. Statistical significance was defined as a p value <0.05. All of the calculations were performed with the Statistical Analysis System software package (version 9.3; Cary, NC, USA).

### Ethics statement

This study was performed in strict accordance with recommendations in the Guide for the Care and Use of Laboratory Animals of the National Institutes of Health. The protocol was approved by the Ethics in Research Committee of the Second Hospital of Jilin University, protocol number 2018–018. Written informed consent was obtained from patients or at least one guardian of the patient before enrolment.

## Supporting information

S1 Table Strains and isolates used in the present study. (DOCX)

S2 Table Clinical data of patients. (DOCX)

S1 Text DNA isolation of clinical specimens. (DOCX)

S1 Fig The LOD and standard curves (Ct vs. log CFU) for *S*. *globosa*, *S*. *schenckii s*. *str* and *S*. *brasiliensis*. (DOCX)
