# Supplementary material for: Fast diagnosis of sporotrichosis caused by Sporothrix globosa, Sporothrix schenckii, and Sporothrix brasiliensis based on multiplex real-time PCR
Source: PLoS Negl Trop Dis. 2019 Feb 28;13(2):e0007219. doi: 10.1371/journal.pntd.0007219 (PMC6394905; doi:10.1371/journal.pntd.0007219)
Supplement: S1 Table — (DOCX) [file pntd.0007219.s001.docx]

S1 Table Strains and isolates used in the present study

| strains | No. | strains | No. |
| --- | --- | --- | --- |
| *Trichophyton rubrum* | BMU00294 | *Sporothrix globosa* | BMU09025 |
| *Trichophyton mentagrophytes* | BMU00293 |  | BMU09026 |
| *Microsporum canis* | BMU00287 |  | BMU09027 |
| *Microsporum gypseum* | BMU00290 |  | BMU09028 |
| *Epidermophyton floccosum* | BMU00286 |  | BMU09029 |
| *Candida albicans* | BMU00260 |  | BMU09030 |
| *Candida tropicalis* | BMU00681 |  | BMU09031 |
| *Candida glabrata* | BMU00678 |  | BMU09032 |
| *Candida parapsilosis* | BMU01732 |  | BMU09033 |
| *Cryptococcus neoformans* | BMU00626 |  | BMU09034 |
| *Aspergillus fumigatus* | BMU00309 |  | BMU09035 |
| *Aspergillus flavus* | BMU00382 |  | BMU09036 |
| *Aspergillus terreus* | BMU00323 |  | BMU09037 |
| *Aspergillus niger* | BMU00614 |  | BMU09038 |
| *Aspergillus nidulans* | BMU00597 |  | BMU09039 |
| *Rhizopus oryzae* | BMU00492 |  | BMU09040 |
| *Exophiala dermatitidis* | BMU00028 |  | BMU09041 |
| *Exophiala jeanselmei* | BMU00457 |  | BMU09042 |
| *Alternaria alternata* | BMU02736 |  | BMU09043 |
| *Cladophialophora carrionii* | BMU13132 |  | BMU09044 |
| *Fonsecaea pedrosoi* | BMU07699 |  | BMU09045 |
| *Phialophoa verrucosa* | BMU01246 | *Sporothrix schenckii s. str* | CBS498.86^T^ |
| *Penicillium marneffei* | BMU02675 |  | BMU08140 |
| *Fusarium solani* | BMU00599 |  | BMU00471 |
| *Fusarium moniliforme* | BMU00713 | *Sporothrix brasiliensis* | CBS 120339^T^ |
| *Pseudallescheria boydii* | BMU07224 |  |  |
| *Scopulariopsis kiliense* | BMU03909 |  |  |
| *Paecilomyces variotii* | BMU05286 |  |  |
| *Escherichia coli* | ATCC25922 |  |  |
| *Staphylococcus aureus* | ATCC29740 |  |  |
| *Staphylococcus epidermidis* | ATCC12228 |  |  |

BMU: Collection of Pathogenic Fungi at Research Centre for Medical Mycology, Peking Universityr

ATCC: American Type Culture Collection

CBS: Centraalbureau voor Schimmelcultures, Utrecht, The Netherlands;

T, type strain
