# Supplementary material for: Fast diagnosis of sporotrichosis caused by Sporothrix globosa, Sporothrix schenckii, and Sporothrix brasiliensis based on multiplex real-time PCR
Source: PLoS Negl Trop Dis. 2019 Feb 28;13(2):e0007219. doi: 10.1371/journal.pntd.0007219 (PMC6394905; doi:10.1371/journal.pntd.0007219)
Supplement: S2 Table — (DOCX) [file pntd.0007219.s002.docx]

S2 Table. Clinical data of patients

| No. | Gender | Age  (year) | Course  (month) | History | | Histopathological examination | Culture | Clinical diagnosis |
| --- | --- | --- | --- | --- | --- | --- | --- | --- |
|  |  |  |  | Trauma | Exposure to contaminants* |  |  |  |
| 1 | F | 39 | 4 | - | + | + | + | Fixed sporotrichosis |
| 2 | M | 40 | 3 | + | + | + | + | Fixed sporotrichosis |
| 3 | F | 58 | 3 | - | + | + | + | Fixed sporotrichosis |
| 4 | M | 36 | 5 | - | + | + | + | Lymphocutaneous sporotrichosis |
| 5 | M | 30 | 2 | - | + | + | + | Lymphocutaneous sporotrichosis |
| 6 | F | 32 | 3 | - | + | + | + | Fixed sporotrichosis |
| 7 | F | 44 | 3 | - | + | + | + | Lymphocutaneous sporotrichosis |
| 8 | F | 61 | 6 | - | + | + | + | Fixed sporotrichosis |
| 9 | M | 67 | 3 | - | + | + | + | Fixed sporotrichosis |
| 10 | F | 8 | 3 | - | + | + | + | Fixed sporotrichosis |
| 11 | F | 37 | 4 | - | + | + | + | Fixed sporotrichosis |
| 12 | M | 40 | 1 | + | + | + | + | Fixed sporotrichosis |
| 13 | M | 45 | 1 | - | + | + | + | Fixed sporotrichosis |
| 14 | M | 70 | 10 | - | + | + | + | Fixed sporotrichosis |
| 15 | F | 82 | 4 | - | - | + | + | Fixed sporotrichosis |
| 16 | F | 56 | 1 | - | - | + | + | Fixed sporotrichosis |
| 17 | M | 64 | 2 | + | + | + | + | Lymphocutaneous sporotrichosis |
| 18 | F | 50 | 3 | + | + | + | + | Fixed sporotrichosis |
| 19 | M | 42 | 2 | + | + | + | + | Lymphocutaneous sporotrichosis |
| 20 | F | 58 | 6 | + | + | + | + | Fixed sporotrichosis |

| 21 | M | 71 | 6 | + | + | + | + | Lymphocutaneous sporotrichosis |
| --- | --- | --- | --- | --- | --- | --- | --- | --- |
| 22 | M | 47 | 1 | + | + | + | + | Fixed sporotrichosis |
| 23 | M | 48 | 12 | - | + | + | + | Fixed sporotrichosis |
| 24 | F | 50 | 4 | + | + | + | + | Lymphocutaneous sporotrichosis |
| 25 | M | 65 | 1 | - | + | + | + | Fixed sporotrichosis |
| 26 | F | 47 | 7 | - | + | + | + | Fixed sporotrichosis |
| 27 | M | 32 | 3 | - | - | + | + | Fixed sporotrichosis |
| 28 | F | 49 | 5 | + | + | + | + | Fixed sporotrichosis |
| 29 | F | 46 | 6 | - | + | + | + | Fixed sporotrichosis |
| 30 | M | 49 | 3 | + | + | + | - ^#^ | Fixed sporotrichosis |
| 31 | M | 60 | 12 | - | + | + | - | Lymphocutaneous sporotrichosis |
| 32 | M | 53 | 4 | + | + | + | -^#^ | Lymphocutaneous sporotrichosis |
| 33 | F | 69 | 2 | - | + | + | - | Fixed sporotrichosis |
| 34 | F | 36 | 3 | - | - | - | - | Mass |
| 35 | F | 62 | 9 | - | - | - | - | Tumor |
| 36 | M | 58 | 6 | - | - | - | - | Keratoacanthoma |
| 37 | M | 29 | 10 | + | - | - | - | Scar |
| 38 | F | 45 | 5 | + | + | - | - | Tumor |
| 39 | F | 23 | 8 | - | - | - | - | Tumor |
| 40 | M | 34 | 6 | + | - | - | - | Tumor |

F:female, M:male; * Mainly corn stalks, mostly used as fuel for heating; ^#^ Contaminated by other microorganism;
