# Supplementary material for: Fast diagnosis of sporotrichosis caused by Sporothrix globosa, Sporothrix schenckii, and Sporothrix brasiliensis based on multiplex real-time PCR
Source: PLoS Negl Trop Dis. 2019 Feb 28;13(2):e0007219. doi: 10.1371/journal.pntd.0007219 (PMC6394905; doi:10.1371/journal.pntd.0007219)
Supplement: S1 Text — (DOCX) [file pntd.0007219.s003.docx]

S1 Text. DNA isolation of clinical specimens

All clinical specimens were tissue biopsies from patients’ skin lesions.

One part of each specimen was cut into small pieces, place all the pieces in liquid nitrogen, grind thoroughly with a mortar and pestle.

Decant tissue powder and liquid nitrogen into 1.5 ml microcentrifuge tube. Allow the liquid nitrogen to evaporate, but do not allow the tissue to thaw, and add 180 μl of Buffer ATL.

Add 20 μl proteinase K, mix by vortexing, and incubate at 56°C until the tissue is completely lysed. Vortex occasionally during incubation to disperse the sample.

Briefly centrifuge the 1.5 ml microcentrifuge tube to remove drops from the inside of the lid.

Add 4 μl RNase A (100 mg/ml), mix by pulse-vortexing for 15 s, and incubate for 2 min at room temperature (15–25°C).

Briefly centrifuge the 1.5 ml microcentrifuge tube to remove drops from inside the lid.

Add 200 μl Buffer AL to the sample. Mix again by pulse-vortexing for 15 s, and incubate at 70°C for 10 min.

Briefly centrifuge the 1.5 ml microcentrifuge tube to remove drops from inside the lid.

Add 200 μl ethanol (96–100%) to the sample, and mix by pulse-vortexing for 15 s.

After mixing, briefly centrifuge the 1.5 ml microcentrifuge tube to remove drops from inside the lid.

Carefully apply the mixture from previous step (including the precipitate) to the QIAamp Mini spin column without wetting the rim.

Close the cap, and centrifuge at 6000 x g (8000 rpm) for 1 min.

Place the QIAamp Mini spin column in a clean 2 ml collection tube and discard the tube containing the filtrate.

Carefully open the QIAamp Mini spin column and add 500 μl Buffer AW1 without wetting the rim.

Close the cap, and centrifuge at 6000 x g (8000 rpm) for 1 min.
Place the QIAamp Mini spin column in a clean 2 ml collection tube and discard the collection tube containing the filtrate.
Carefully open the QIAamp Mini spin column and add 500 μl Buffer AW2 without wetting the rim.

Close the cap and centrifuge at full speed (20,000 x g; 14,000 rpm) for 3 min.

Place the QIAamp Mini spin column in a new 2 ml collection tube and discard the old collection tube with the filtrate. Centrifuge at full speed for 1 min. Place the QIAamp Mini spin column in a clean 1.5 ml microcentrifuge tube, and discard the collection tube containing the filtrate.

Carefully open the QIAamp Mini spin column and add 100 μl Buffer AE. Incubate at room temperature for 1 min, and then centrifuge at 6000 x g (8000 rpm) for 1 min, and repeat this step.

Transfer the AE, which is containing the DNA, to a new 1.5 ml microcentrifuge tube and store at -80 °C until use.
