# Supplementary material for: Fast diagnosis of sporotrichosis caused by Sporothrix globosa, Sporothrix schenckii, and Sporothrix brasiliensis based on multiplex real-time PCR
Source: PLoS Negl Trop Dis. 2019 Feb 28;13(2):e0007219. doi: 10.1371/journal.pntd.0007219 (PMC6394905; doi:10.1371/journal.pntd.0007219)
Supplement: S1 Fig — (DOCX) [file pntd.0007219.s004.docx]

S1 Fig. The LOD and standard curves (Ct vs. log CFU) for *S.globosa, S. schenckii s. str* and *S. brasiliensis*.


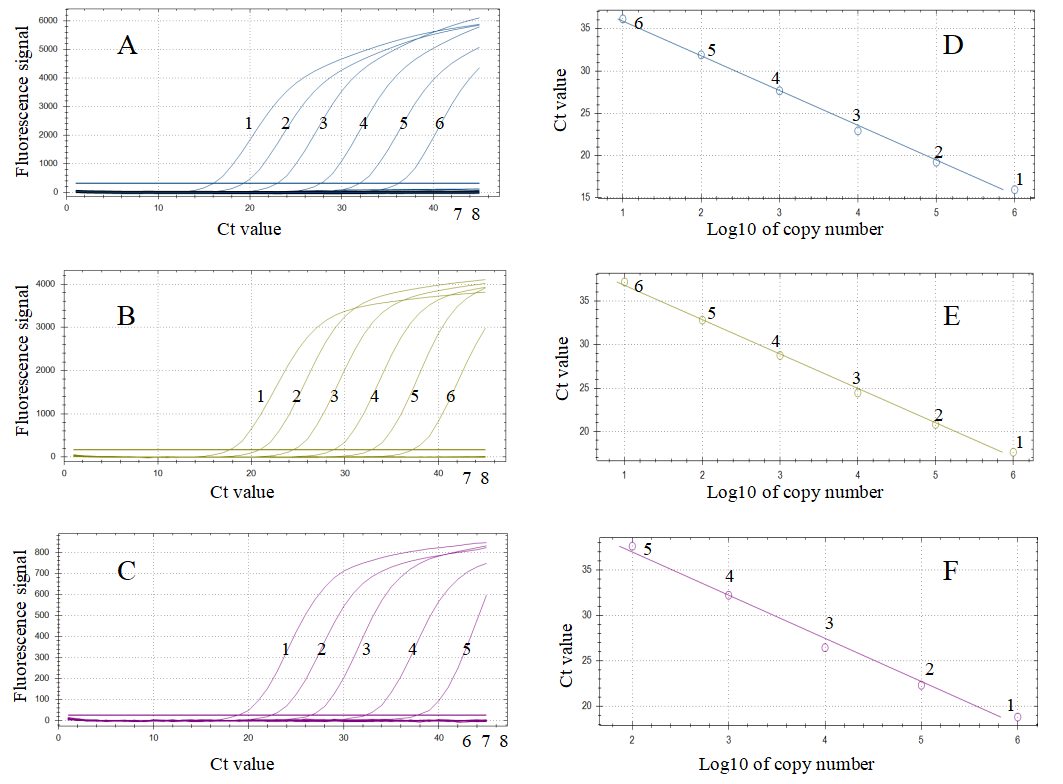


1：10^6^copies，2：10^5^copies，3：10^4^copies，4：10^3^copies，5：10^2^copies，6：10copies，7：1 copies，8：NTC

A,D: *S.globosa*, B,E: *S. schenckii s. str*, C,F:*S. brasiliensis*; A,B,C: LOD, D,E,F: standard curves
